# LncRNA PITPNA-AS1 mediates the diagnostic potential of miR-129-5p in prostate cancer

**DOI:** 10.1186/s12894-024-01528-2

**Published:** 2024-07-13

**Authors:** Zhaolu Song, Silei Xu, Xiaohui Gu, Qiang Feng, Chang Wang

**Affiliations:** 1 Department of Urology Surgery, Jiaozhou Central Hospital of Qingdao, Shandong, 266300 China; 2 Medical School of University of Electronic Science and Technology of China, Chengdu, 610051 China; 3 Department of Urinary Surgery, Sichuan Academy of Medical Sciences, Sichuan Provincial People’s Hospital, No. 32, West Section 2, 1st Ring Road, Qingyang District, Chengdu, 610031 China; 4 Organ Transplant Center, The First Affiliated Hospital, Sun Yat-sen University, No. 58, Zhongshan Second Road, Guangzhou City, 510080 Guangdong Province China

**Keywords:** lncRNA PITPNA-AS1, Prostate cancer, miR-129-5p, Diagnosis, Tumor progression

## Abstract

**Background:**

LncRNA has an effective value in many diseases, which has long been applied in the diagnosis, treatment and prognosis of prostate cancer. This study focused on lncRNA PITPNA-AS1, and its diagnostic potential in prostate cancer has been explored.

**Methods:**

The expression of PITPNA-AS1 and miR-129-5p in prostate cancer serum and sample cells was determined by real-time quantitative polymerase chain reaction (RT-qPCR). The relationship between the expression of PITPNA-AS1 and clinicopathological parameters was considered. ROC curve prompted the diagnostic value of PITPNA-AS1. The effect of PITPNA-AS1 on prostate cancer cells was verified using vitro cells assay. Luciferase activity assay and RIP assay demonstrated the sponge relationship of PITPNA-AS1 to miR-129-5p.

**Results:**

PITPNA-AS1 level was increased, while miR-129-5p was obviously decreased in prostate cancer. PITPNA-AS1 expression was associated with Gleason grade, lymph node metastasis and TNM stage in patients. The area under the curve (AUC) was 0.910, with high sensitivity and specificity. PITPNA-AS1 was elucidated to directly target miR-129-5p, whereas silencing PITPNA-AS1 negatively affected prostate cancer cell proliferation, migration and invasion. Intervention of miR-129-5p inhibitor reversed the effect of silencing PITPNA-AS1 on cells.

**Conclusions:**

PITPNA-AS1 was relatively highly expressed in prostate cancer and mediated the pathophysiological process of patients, which may serve as a diagnostic indicator. Silencing of the PITPNA-AS1 sponge miR-129-5p inhibited the biological function of the cells, indicating that PITPNA-AS1 may represent a novel therapeutic target for prostate cancer.

**Supplementary Information:**

The online version contains supplementary material available at 10.1186/s12894-024-01528-2.

## Background

Prostate cancer is a malignant tumor of male urinary system with insidious onset [1]. Most patients are already in advanced stage when diagnosed, which seriously threatens the life and health of male patients [2]. With changes in diet and environment, morbidity and mortality increase rapidly, especially in economically developed regions. Currently, the mechanisms of occurrence and metastasis of prostate cancer are still unclear, and the clinical-pathological mechanism of prostate cancer is lack unified understanding, which also causes the delay in diagnosis [3]. Therefore, it is particularly urgent to understand the physiological mechanism of prostate cancer in detail and to search the diagnostic markers.

Long non-coding RNAs (LncRNAs) have been shown to regulate diseases through various pathways, exerting extensive influence on the growth, development and differentiation of patient cells [4]. For example, lncRNA LUCAT1 was associated with a variety of tumors, including lung cancer, and induced tumor expression and progression [5]. LncRNA HOXA-AS2 was aberrantly expressed in many human cancers as well, directly or indirectly mediating the expression of target genes [6]. Furthermore, LINC00673 [7], HOTAIR [8], and CASC9 [9] have also carried out related tumor studies. These records further suggest that the differential expression of lncRNAs in tumors has high research value.

According to the literatures, PITPNA-AS1 was a promising therapeutic marker for regulating malignant progression of lung squamous cell carcinoma [10]. In the exploration of gastric cancer, PITPNA-AS1 played a role in targeting miR-98-5p, which may be used as a potential therapeutic target [11]. Guo et al. demonstrated that PITPNA-AS1 mediated the cycle and apoptosis of cervical cancer cells and thereby promoted the progression of cervical cancer through sponge miR-876-5p [12]. Moreover, PITPNA-AS1 was also involved in the development of glioblastoma [13], hepatocellular carcinoma [14], triple-negative breast cancer [15], whereas, its application in prostate cancer has not been discussed yet and deserves further study. In previous studies of prostate cancer, the lncRNA NEAT1 sponge miR-766-5p/E2F3 axis accelerated disease progression [16]. SNHG1 binding to miR-383-5p enhanced prostate cancer progression [17]. These results suggest that microrRNAs (miRNAs) may interact with lncRNAs to influence the generation and development of tumors. Therefore, we focused on the possibility of PITPNA-AS1 as a diagnostic factor and the impact of lncRNA-miRNA on the progression of prostate cancer in this study.

## Materials and methods

### General data

A total of 112 newly diagnosed prostate cancer patients and 80 healthy controls who received treatment in Sichuan Academy of Medical Sciences, Sichuan Provincial People’s Hospital from March 2020 to March 2022 were included in this study, and the whole process was conducted under the supervision of the Hospital Ethics Committee (No. 2019–229). Inclusion criteria: newly diagnosed patients with prostate cancer; patients who have not received antitumor therapy before; patients and their families agree to cooperate with this study. Exclusion requirements: patients with other malignant tumors; patients with secondary prostate cancer; patients with cardiovascular and cerebrovascular, diabetes or infectious diseases; patients who failed to sign informed consent. General information such as age, tumor size, and PSA level of the participating prostate cancers were collected and recorded. Meanwhile, lymph node metastasis refers to the process of prostate cancer cells spreading through the lymphatic system to other parts of the body. This is a sign of the aggressiveness of prostate cancer, which means that the cancer may have spread beyond the prostate itself to other parts of the body. We evaluated by imaging examination and histopathological examination, and PSMA PET/CT can be used as a means of auxiliary diagnosis.

### Collection of blood specimens and tissue samples

Blood specimens were collected from all participants with the knowledge and consent of all participants, and serum was separated in time. The obtained serum samples were stored in a refrigerator at -80 °C for later use.

In addition, 54 prostate cancer tissue samples and paracancerous normal tissue samples were washed with PBS and similarly cryopreserved.

### Purchase and culture of cells

Cell samples including prostate cancer cells (DU145, 22Rv1, LNCaP and PC3) and normal prostate cells RWPE-1 were purchased from ATCC in the United States, and they were inoculated in RPMI 1640 medium (Sigma-Aldrich, USA) supplemented with 10% inactivated fetal bovine serum (FBS). All cells were incubated at 37 °C and maintained in an incubator containing 5% CO_2_.

### Evaluation of PITPNA-AS1 and miR-129-5p expression

After predicting the PITPNA-AS1 and miR-129-5p contents in prostate cancer using the ENCORI database, their levels were determined in the serum samples collected in this study. Total RNA was extracted from serum samples with Trizol reagent. The concentration and purity of RNA were measured in time to determine the quality of RNA. Total RNA as template, cNDA was synthesized according to TIANGEN reverse transcription kit (Beijing, China). SYBR Green fluorescent polymerase chain reaction kit (Thermo Fisher, USA) was used to configure the reaction system, and RT-qPCR was performed with 7500 FAST PCR instrument (ABI, USA). Using GAPDH and U6 as internal parameters, the relative expression levels of PITPNA-AS1 and miR-129-5p in the samples to be tested were calculated through 2^−ΔΔCT^ method.

### Cell transfection and in vitro assay

The control group, silencing negative control (si-NC) and silencing PITPNA-AS1 (si-PITPNA-AS1) were transfected into prostate cancer cells LNCaP and PC3 in the presence of Lipofectamine 2000 reagent (Invitrogen, USA). Simultaneously, the transfection results were reflected by the relative expression of PITPNA-AS1.

Transfected cells (si-PITPNA-AS1-LNCaP and si-PITPNA-AS1-PC3) were cultured in 96-well plates under the same conditions as above. CCK-8 reagent was added at appropriate times and culture was continued, then OD value was detected 2 h later to understand the proliferation activity of cells. Transfected cells LNCaP and PC3 were inoculated in the upper chamber of the Transwell plate for cell metastasis assay, in which Matrigel-coated upper chambers were used for invasion level detection, and non-coated upper chambers were used for migration experiment. The cells were transferred to an upper chamber filled with RPMI-1640 medium, while the lower chamber was filled with RPMI-1640 medium supplemented with 10% FBS. The cells were incubated and calculated after crystal violet staining.

### Western blot assay of cell markers

The expression of interstitial markers vimentin and N-cadherin and epithelial adhesion marker E-cadherin were detected by Western blot. Cells were cultured in 6-well plates for 48 h and then lysed by protein extraction kit (Biyuntian, China). Protein samples were added to SDS-PAGE gels and transferred to polyvinylidene difluoride membranes. Membranes were applied to antibody solutions for vimentin, N-cadherin, E-cadherin, and GAPDH, and peroxidase coupled secondary antibodies were added and incubated for 2 h. Protein bands were quantified and visualized using a luminescence image analyzer and ImageJ 1.42q software.

### RIP assay

RIP assay was carried out with IgG antibody and Ago2-specific, and RNA in PC3 cells was extracted by RIP RNA-Binding Protein Immunoprecipitation Kit (Millipore, USA). The interaction between PITPNA-AS1 and miR-129-5p was reflected according to the relative content of PITPNA-AS1.

### Dual-luciferase reporter assay

The binding sites of PITPNA-AS1 and miR-129-5p were predicted by LncBook 2.0 database and then confirmed using luciferase activity assay. PC3 cells were cultured in 48-well plates to cell adherence, and then co-transfected with the control group, mimic NC, inhibitor NC, miR-129-5p mimic or miR-129-5p inhibitor and PITPNA-AS1-WT (wild-type) or PITPNA-AS1-MUT (mutant-type) with Lipofectamine 2000 reagent. Correlation analysis was performed with relative luciferase activity as reference.

### Statistical analysis

After each experiment was repeated three times, data statistics were performed by SPSS 21.0 software. The enumeration data were tested by chi-square test, and the measurement data were expressed as mean ± standard deviation. The diagnostic value of PITPNA-AS1 was assessed by ROC curve, and the prediction accuracy is higher when AUC > 0.9.

## Results

### The relative expression quantity of PITPNA-AS1 and miR-129-5p in prostate cancer

**Fig. 1:**
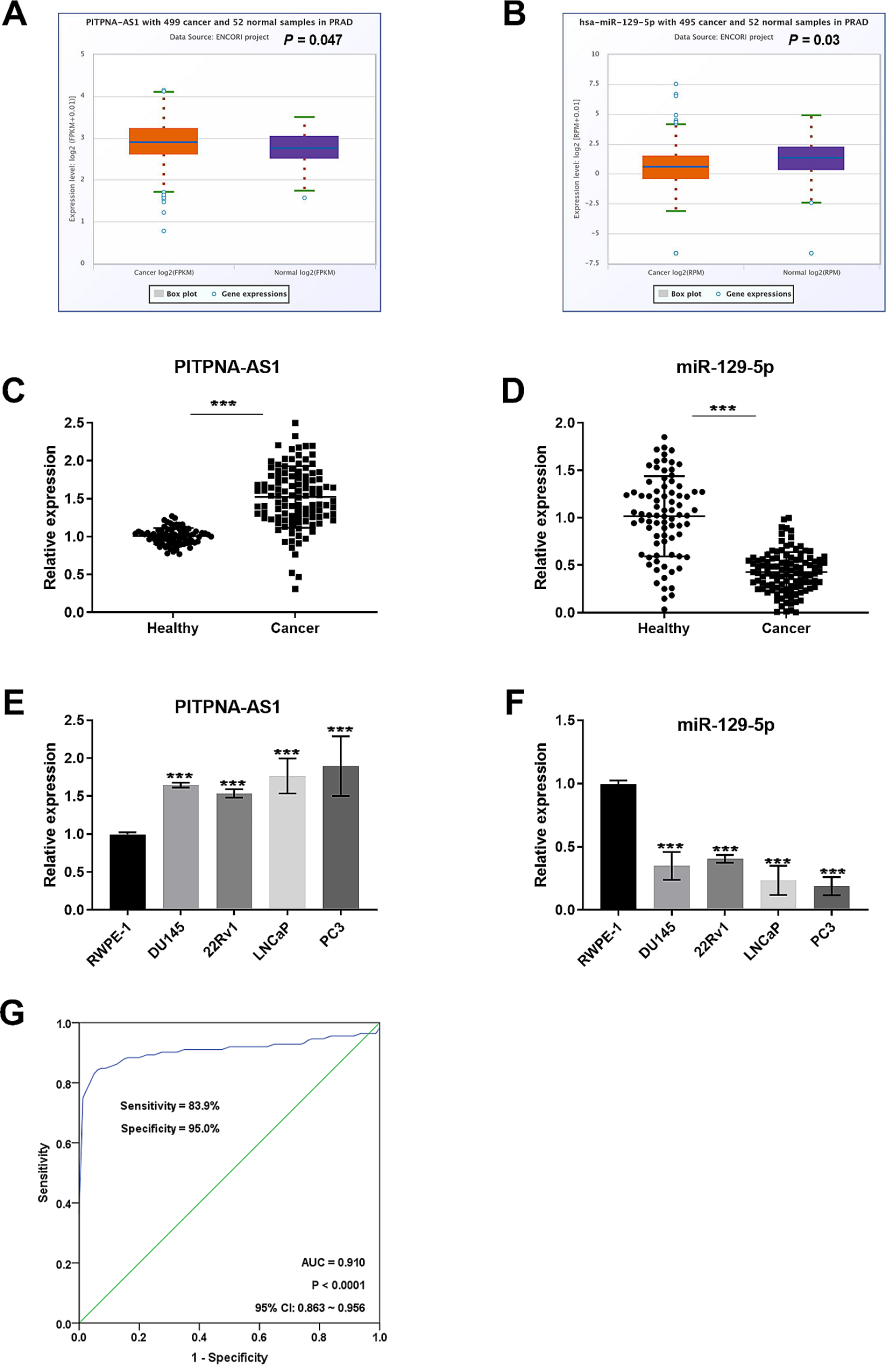
The relative expression quantity of PITPNA-AS1 and miR-129-5p in prostate cancer. (**A** and **B**) The expression trend of PITPNA-AS1 and miR-129-5p in prostate cancer samples was predicted by ENCORI database. (**C**) Elevated expression of PITPNA-AS1 in prostate cancer serum. (**D**) miR-129-5p was downregulated in tumor serum compared with healthy controls. (**E** and **F**) PITPNA-AS1 was elevated while miR-129-5p was down-regulated in prostate cancer cells. (**G**) ROC curves illustrating the diagnostic value of PITPNA-AS1 in prostate cancer (AUC = 0.910, *P* < 0.0001, 95% CI: 0.863 ∼ 0.956). ***P < 0.001

According to ENCORI online database analysis, PITPNA-AS1 expression in prostate cancer samples was prominently expressed in prostate cancer samples compared with normal samples, while the miR-129-5p level was decreased (Fig. 1A and B). In addition, PITPNA-AS1 in prostate cancer serum was higher than that in healthy controls (Fig. 1C). On the contrary, the expression of miR-129-5p was decreased in cancer serum (*P* < 0.001, Fig. 1D). The expression of PITPNA-AS1 and miR-129-5p was detected in cells, and it was also found that PITPNA-AS1 was obviously upregulated in prostate cancer cells, while miR-129-5p was downregulated (Fig. 1E and F). Notably, PITPNA-AS1 was prominently expressed in prostate cancer tissues and miR-129-5p was poorly expressed as determined by RT-qPCR assays in 54 prostate cancer tissue samples collected (Supplementary Figure).

### The value of PITPNA-AS1 in the diagnosis of prostate cancer patients

The relationship between PITPNA-AS1 expression and clinical characteristics in prostate cancer serum was compared and analyzed. Table 1 describes that PITPNA-AS1 expression were not related to the age, tumor size, and PSA of patients (*P* > 0.05), which was correlated with Gleason grade, lymph node metastasis and TNM stage (*P* < 0.05). Furthermore, ROC curves were drawn to evaluate the diagnostic performance of PITPNA-AS1 in prostate cancer patients. According to Fig. 1G, the AUC of PITPNA-AS1 in identifying prostate cancer patients was 0.910, with the sensitivity of 83.9% and the specificity of 95.0% (*P* < 0.0001, 95% CI: 0.863 ∼ 0.956). The above stated that PITPNA-AS1 has a high diagnostic value in distinguishing prostate cancer patients from healthy individuals.

**Table 1:** Clinical characteristics of patients with prostate cancer

Parameters	lncRNA PITPNA-AS1 expression		*P*
	Low (*n* = 55)	High (*n* = 57)	
Age			0.638
≤ 60	38	37	
> 60	17	20	
Gleason grade			0. 028
≤ 7	40	30	
> 7	15	27	
Tumor size			0.462
≤ 3 cm	25	22	
> 3 cm	30	35	
PSA (ng/mL)			0.464
≤ 4	23	20	
> 4	32	37	
Lymph node metastasis			0.044
Negative	40	31	
Positive	15	26	
TNM stage			0.008
I-II	37	24	
III-IV	18	33	

### Regulation of LNCaP and PC3 cells by silencing PITPNA-AS1

**Fig. 2:**
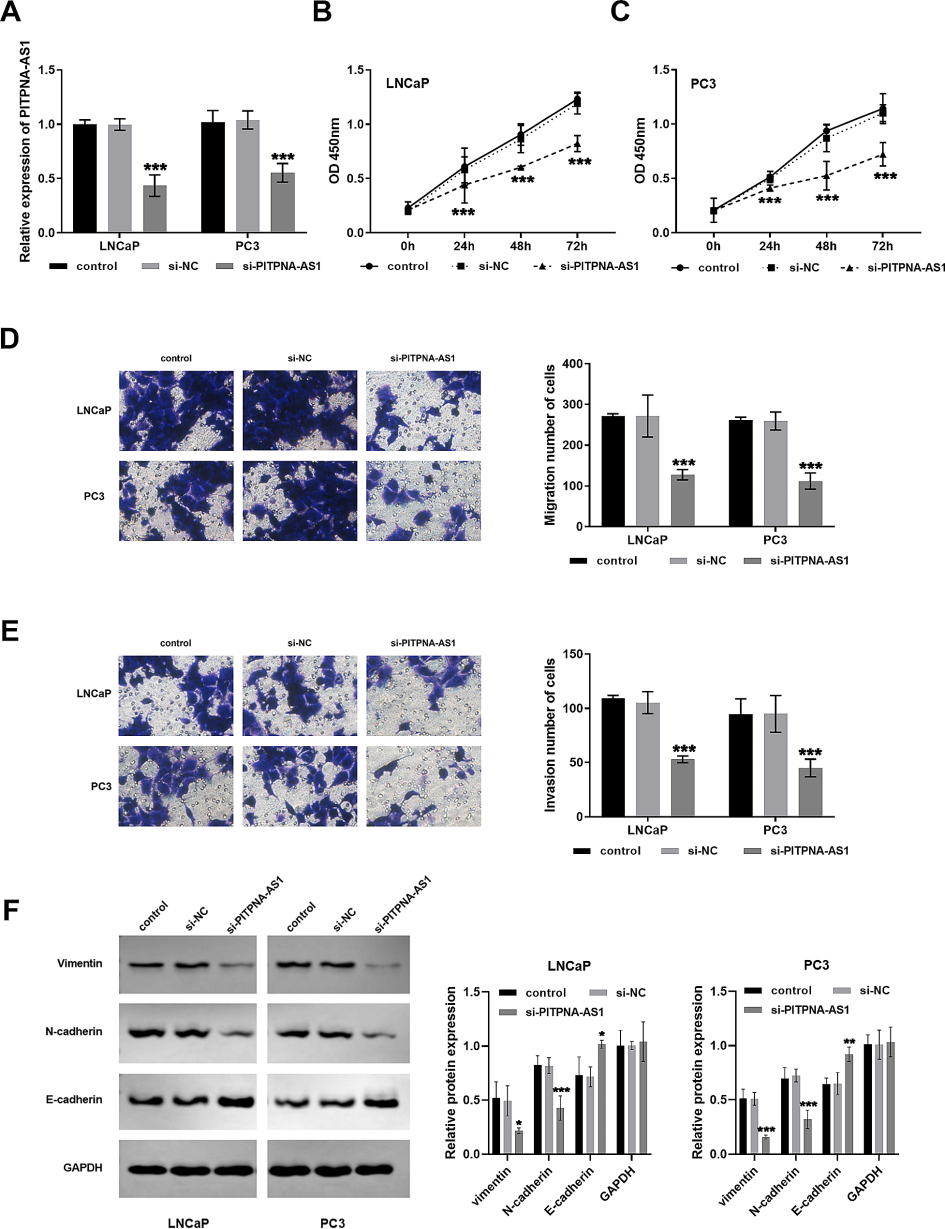
Regulation of silencing PITPNA-AS1 in prostate cancer cells. (**A**) Transfection of knockdown PITPNA-AS1 in LNCaP and PC3 cells. (**B** and **C**) Knockdown of PITPNA-AS1 slowed the proliferation level of cells. (**D**) Effects of knockdown of PITPNA-AS1 on cell migration. (**E**) Silencing of PITPNA-AS1 repressed the invasive capacity of cells. (**F**) Western blot analysis after silencing PITPNA-AS1 transfection. *P < 0.05, **P < 0.01, ***P < 0.001

Based on the fact that PITPNA-AS1 was elevated obviously in LNCaP and PC3 cells, they were selected for in vitro cell assays. After transfection with si-PITPNA-AS1, the level of PITPNA-AS1 in the cells was reduced (Fig. 2A). The effect of PITPNA-AS1 knockdown on the proliferation of prostate cancer cells was detected by CCK-8 method. Figure 2B and C indicates that compared with the control group, OD 450 nm decreased after PITPNA-AS1 knockdown, indicating that cell proliferation level was inhibited. In Fig. 2D and E, knockdown PITPNA-AS1 also repressed the migration and invasion ability of LNCaP and PC3 cells. Additionally, vimentin and N-cadherin decreased, while E-cadherin increased after transfection with si-PITPNA-AS1 (Fig. 2F).

### Interaction of PITPNA-AS1 and miR-129-5p

**Fig. 3:**
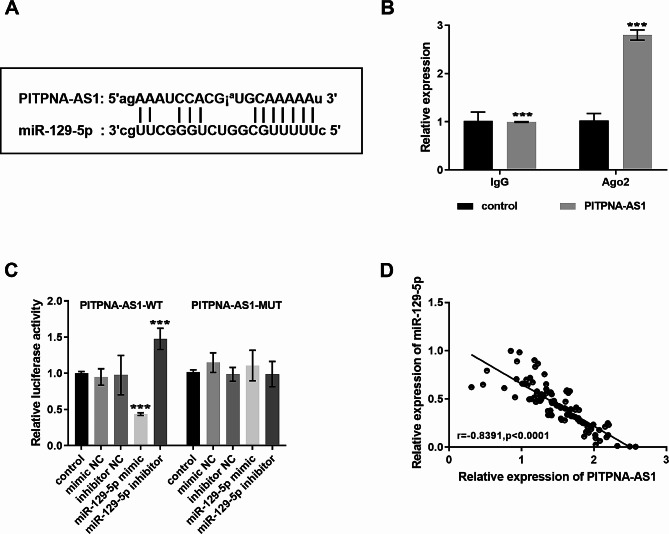
Interaction of PITPNA-AS1 and miR-129-5p. (**A**) There are binding sites between PITPNA-AS1 and miR-129-5p. (**B**) Ago2 antibody significantly enriched PITPNA-AS1. (**C**) The luciferase activity assay suggested that PITPNA-AS1 directly targeted miR-129-5p. (**D**) PITPNA-AS1 and miR-129-5p were negatively correlated (*r* = -0.8391, *P* < 0.0001). ***P < 0.001

Through sequence alignment analysis, it was found that the PITPNA-AS1 sequence had binding sites with miR-129-5p, as shown in Fig. 3A. RIP assay were conducted in prostate cancer cells, and the results showed that Ago2 antibody could significantly enrich PITPNA-AS1, suggesting that PITPNA-AS1 could interact with Ago2 (Fig. 3B), which indirectly proved that PITPNA-AS1 had the ability to bind miR-129-5p. PITPNA-AS1 may act as a molecular sponge to adsorb miR-129-5p. To verify the targeting effect of PITPNA-AS1 on miR-129-5p, luciferase reporter gene detection was performed. The luciferase activity was weakened after co-transfection of miR-129-5p mimic and PITPNA-AS1-WT, while co-transfection of miR-129-5p inhibitor and PITPNA-AS1-WT enhanced the luciferase activity. Furthermore, the involvement of PITPNA-AS1-MUT had no significant effect on luciferase activity, confirming that PITPNA-AS1 directly targeted miR-129-5p (Fig. 3C). Subsequently, the correlation analysis between PITPNA-AS1 and miR-129-5p showed that the correlation coefficient was -0.8391 in Fig. 3D, showing a significant negative correlation.

### Regulation of miR-129-5p inhibitor on prostate cancer cells

**Fig. 4:**
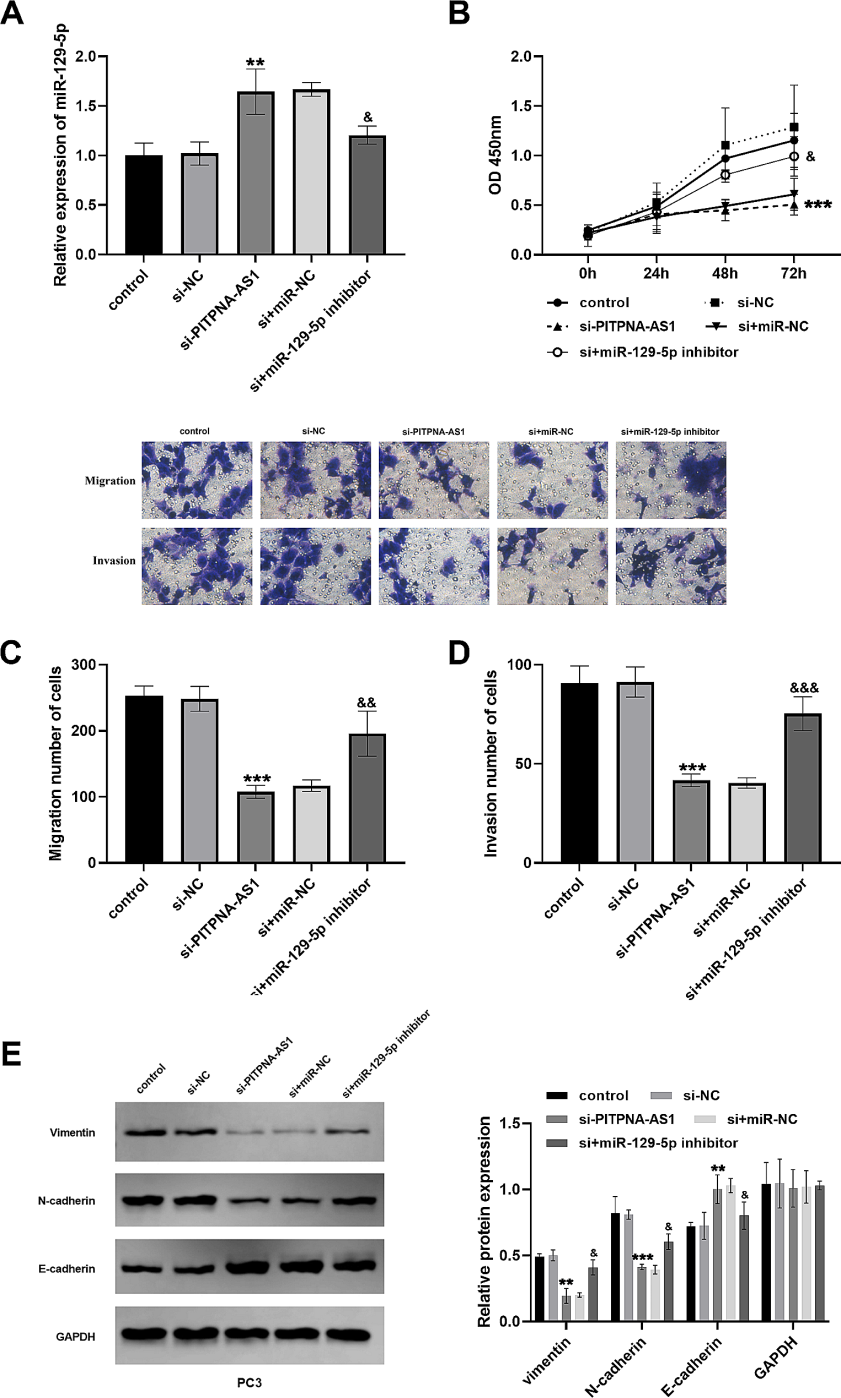
Regulation of miR-129-5p inhibitor on prostate cancer cells. (**A**) Expression of miR-129-5p in cells transfected with different indexes. (**B**-**D**) Transfection of miR-129-5p inhibitor reversed the biological function of prostate cancer cells. (**E**) Western blot analysis after co-transfection with silencing PITPNA-AS1 and miR-129-5p inhibitor. **P < 0.01, ***P < 0.001, vs. control; ^&^*P* < 0.05, ^&&^*P* < 0.01, ^&&&^*P* < 0.001, vs. si-PITPNA-AS1

Transfection silencing of PITPNA-AS1 elevated miR-129-5p levels in PC3 cells, whereas the involvement of miR-129-5p inhibitor restored miR-129-5p level (Fig. 4A). Silencing of PITPNA-AS1 suppressed the growth ability of prostate cancer cells, whereas downregulation of miR-129-5p restored cell growth activity (Fig. 4B). Meanwhile, downregulation of PITPNA-AS1 repressed the biological functions of the cells, while the migratory (Fig. 4C) and invasive (Fig. 4D) abilities of the cells were reversed after transfection with si + miR-129-5p inhibitor. Meanwhile, the levels of vimentin and N-cadherin proteins were increased, while the E-cadherin protein level was decreased after treatment with si-PITPNA-AS1 and miR-129-5p inhibitor (Fig. 4E).

## Discussion

Prostate cancer is a kind of tumor caused by malignant hyperplasia of prostate epithelial cells, which is not infectious, but with a certain degree of heritability. Age is the biggest risk factor for prostate cancer, and obesity, diet and sexual activity also increase the risk [18]. In the early stage of the disease, the tumor grows slowly, and most patients have no obvious symptoms. Once the tumor causes prostate swelling or spreads outside the prostate, it may lead to frequent urination, hematuria accompanied by obvious pain, and even lead to bone pain or fracture [19]. Based on this, timely diagnosis and treatment of prostate cancer is very urgent, which may effectively alleviate the suffering of patients.

LncRNAs intervene in various processes such as regulation of transcription and translation, regulation of cell cycle, apoptosis and chromatin modification. Additionally, a large number of studies have shown that LncRNAs are abnormally expressed in a variety of malignant tumors and play a key regulatory role, which are closely related to the occurrence and development of tumors and have the potential as markers for diagnosis, treatment and prognosis of patients [20]. PITPNA-AS1 is located on chromosome 17p13.3 and is prominently expressed in non-small cell lung cancer, whereas silencing PITPNA-AS1 suppresses cell growth and metastasis [21]. Yuan and colleagues claimed that silencing PITPNA-AS1 accelerated the apoptosis of colorectal cancer cells, which adsorption of miR-129-5p inhibited tumor progression [22]. In this study, PITPNA-AS1 was determined to be overexpressed in prostate cancer, and proliferation, migration and invasion of prostate cancer cells were attenuated when PITPNA-AS1 was knocked down. In addition, elevated PITPNA-AS1 expression predisposed patients with lung squamous cell carcinoma [23] and gastric cancer [24] to develop fatal cancers, which was consistent with our reported results. The abnormal expression of PITPNA-AS1 in prostate cancer serum was affected by Gleason grade, lymph node metastasis, and TNM stage, suggesting that PITPNA-AS1 may be involved in the occurrence of prostate cancer. Moreover, ROC curves analysis showed that PITPNA-AS1 had a high predictive significance in prostate cancer, implying that PITPNA-AS1 may be a new marker for clinical evaluation of prostate cancer.

MiRNA is a non-coding small molecule single-stranded RNA, which is the main element regulating gene translation level and transcription level expression [25]. And studies have confirmed that miRNAs or binding lncRNAs are involved in the pathophysiological processes of tumors, including cell proliferation, apoptosis, apoptosis tumorigenesis, and metastasis [26]. In the miRNA study of prostate cancer, miR-32-5p, miR-20a-5p and miR-454-3p were downexpressed, which are reference diagnostic markers [27]. Significantly reduced miR-21 and miR-100 improve predictive levels of prostate cancer and also have the potential to be diagnostic markers [28]. miR-129-5p was reduced in prostate cancer was revealed in this study, and both RIP assay and luciferase activity assay confirmed that miR-129-5p was the direct target of PITPNA-AS1. In previous evidence, Luo et al. elucidated that lncRNA RP11-89 sponges miR-129-5p to promote bladder carcinogenesis [29] and Wu et al. described that lncRNA HOTAIR regulates the miR-129-5p/FZD7 axis to accelerate breast cancer expansion [30]. Meanwhile, miR-129-5p binds downstream DLX1 [31], ETV1 [32] and CAMK2N1 [33] targets to mediate the progression of prostate cancer and affect the biological functions of cancer cells. Furthermore, miR-129-5p inhibitor partially restored the suppressive effect of PITPNA-AS1 knockdown on the proliferation and activity of prostate cancer cells, and this was also indicated by Western blot assay of cellular markers. This implies that PITPNA-AS1 may play a regulatory role in prostate cancer through sponge miR-129-5p.

The above studies revealed that PITPNA-AS1 is enriched in prostate cancer, while miR-129-5p expression is dysregulated. Downregulation of PITPNA-AS1 directly sponge miR-129-5p to mediate the deterioration of prostate cancer. What is noteworthy is that the small sample size of prostate cancer and lack of in vivo cell experiments are the limitations of this study, which need to be tested by large samples and more detailed studies in the future.

## Conclusions

In general, PITPNA-AS1 was abnormally overexpressed in prostate cancer and had a potential as a diagnostic marker. Knockdown of PITPNA-AS1 targeting miR-129-5p may suppress tumor progression, providing a possible therapeutic target for prostate cancer.

### Electronic supplementary material

Below is the link to the electronic supplementary material.

Supplementary Material 1

Supplementary Material 2

## Data Availability

Corresponding authors may provide data and materials.

## Abbreviations

AUC: Area under the curve
RT-qPCR: Real-time quantitative polymerase chain reaction
