# Supplementary figures and images for: LncRNA PITPNA-AS1 mediates the diagnostic potential of miR-129-5p in prostate cancer
Source: BMC Urol. 2024 Jul 13;24:146. doi: 10.1186/s12894-024-01528-2 (PMC11245843; doi:10.1186/s12894-024-01528-2)

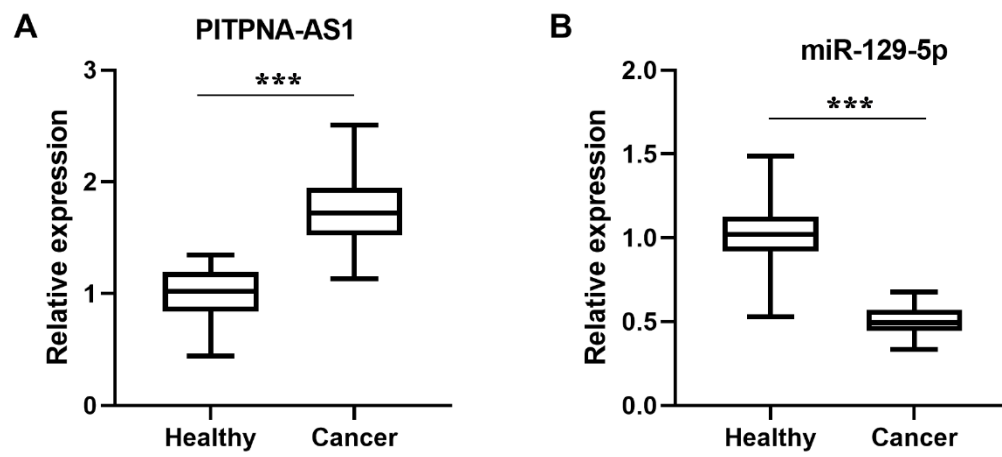

**Supplementary Figure.** Detection of (A) PITPNA-AS1 and (B) miR-129-5p levels in prostate cancer tissues. \*\*\* $P < 0.001$ .

Supplement: Supplementary file 1 — Supplementary Material 1 [file 12894_2024_1528_MOESM1_ESM.pdf]
