# Supplementary material for: LncRNA PITPNA-AS1 mediates the diagnostic potential of miR-129-5p in prostate cancer
Source: BMC Urol. 2024 Jul 13;24:146. doi: 10.1186/s12894-024-01528-2 (PMC11245843; doi:10.1186/s12894-024-01528-2)
Supplement: Supplementary file 2 — Supplementary Material 2 [file 12894_2024_1528_MOESM2_ESM.pdf]

Supplementary Info File

Figure 2. (F)

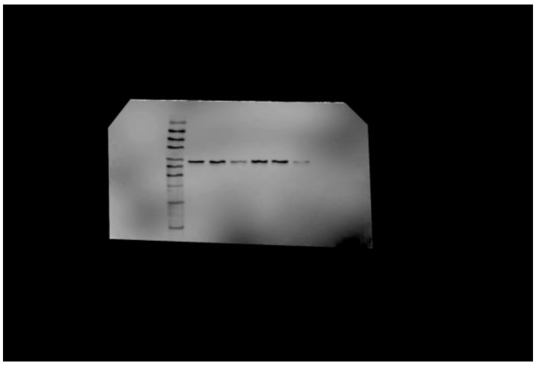

Vimentin

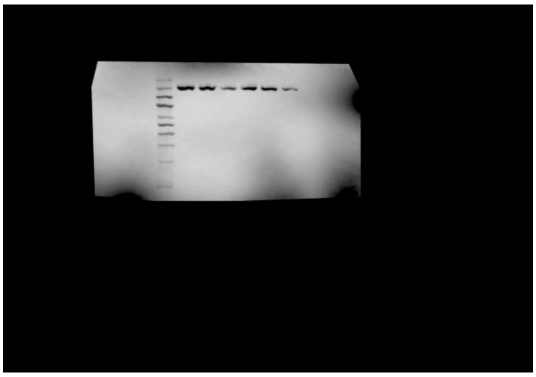

N-cadherin

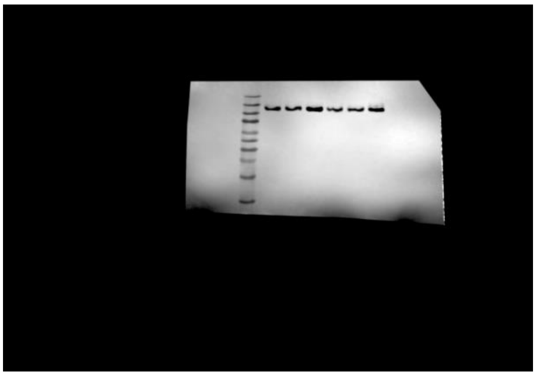

E-cadherin

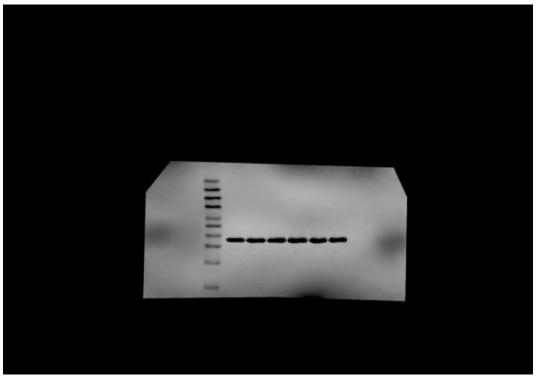

GAPDH

Figure 4. (E)

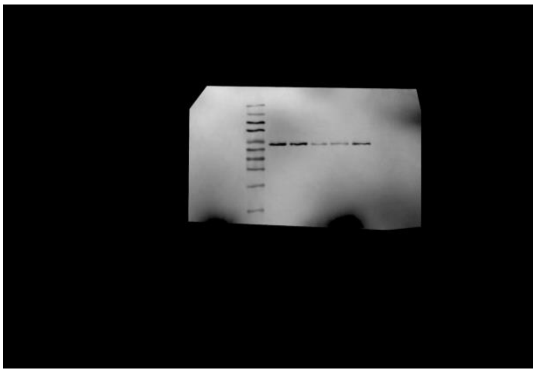

Vimentin

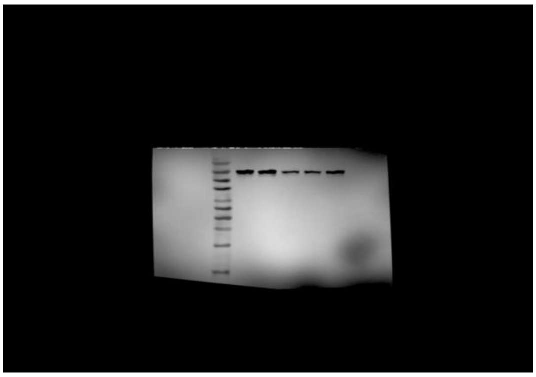

N-cadherin

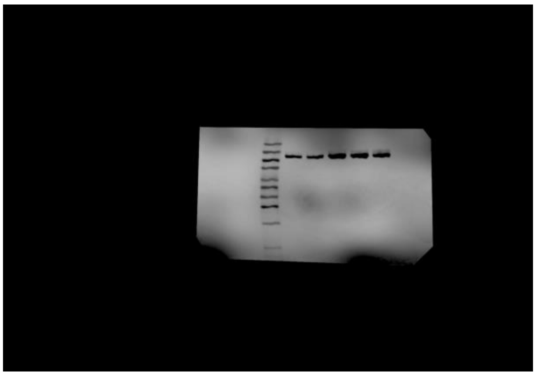

E-cadherin

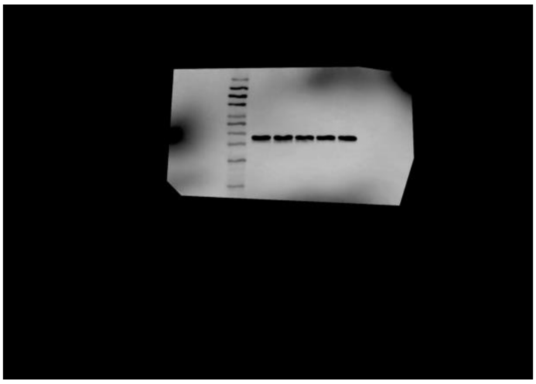

GAPDH
